# Digital risk distribution and COVID-19: How contact tracing is promoted as a solution to equilibrate public health and economic prosperity during pandemics

**DOI:** 10.1177/20552076221085068

**Published:** 2022-03-18

**Authors:** Dana Mahr, Marylaure Bloch

**Affiliations:** 1 30500 Université de Genève Faculté des Sciences, Geneve, Switzerland; 2 Institut Confucius, 151067 Université de Genève Faculté des lettres, Pregny-Chambésy, Switzerland

**Keywords:** Severe acute respiratory syndrome-coronavirus-2, proximity tracing, eHealth, general, smartphone, media, policy

## Abstract

Digital contact tracing appears as an ideal solution to tackle long-term economic damage due to necessary lockdown measures during a pandemic. This essay shows that the challenge of balancing citizen's health and a functioning society is not just coming up today. Commercial centres were already in the Middle Ages worried about their economic prosperity and adopted isolation measures. Although there are much more data available today, pandemic preparedness remains constrained by temporal and spatial realities, thus limiting public health management to the national state. Based on the examples of China and Switzerland, we elaborate on how individual and collective needs can be balanced differently regarding the implementation of a digital contact tracing system. While China's Health Code App is close to social surveillance, Switzerland has turned away from Europe to develop its own Swiss solution due to disagreement about data protection. It becomes clear that the attempts to properly balance public health and economic prosperity during a pandemic must be constantly readjusted and cannot simply be delegated to a digital technology.

## Introduction

Health-oriented smartphone apps have become a vital part of our everyday lives. We use them to track and predict our menstrual cycles, to add up the calories we consume, to count the steps we take every day and so much more. Now they promise us also a way out of the COVID-19 crisis. Their function involves an algorithm-based evaluation of our movement patterns and social contacts, while their user interface resembles a real-world version of Harry Potter's fictional Marauder's map.^1^

During the ongoing COVID-19 pandemic, governments all over the world adopted public health strategies of different rigour, yet all with a certain awareness that there is a thin line between protecting the citizens and damaging the country's economic prosperity.^2^ As soon as the infection rates dropped; governments started planning the exit out of the lockdown.^3^ Low infection rates allowed public health officials to rely on (digital) contact tracing as a means of pandemic prevention.^4^ The use of Apps collecting and mapping people's mobility seemed a promising option for many political, medical, and industrial actors to ensure a nationwide contact tracing.^5^ Although since the beginning of the use of contact tracking apps, especially in Europe and the USA, disillusionment has spread with regard to the reliability of the collected data material,^6^ there have also been epidemiological success stories,^7^ especially from Southeast Asian societies,^8^ above all the Chinese special administrative zones of Taiwan and Hong Kong with their compact territories.^9^ Despite ethical issues and questions regarding the quality of tracking data, many stakeholders from politics, science, and society are still very positive about the use of corresponding applications in the third year of the pandemic.^10^

From both a sociological and historical perspective, severe acute respiratory syndrome-coronavirus-2 (SARS-CoV-2) related contact tracing Apps appear to meet two needs. On the one hand, they allow resuming economical and other activities, while fulfilling the state's duty to protect the health of the collective. On the other hand, contact tracing Apps operate at the level of the individual as a self-regulatory tool to generate a feeling of safety in a time of uncertainty. Both the collective and the individual needs are embedded in a fragile balance of control. The aim of this article is to reflect on this delicate balance between disease containment for the good of the citizens and total surveillance. The first section turns towards the origins of pandemic-related public health measures by illustrating how authorities dealt with the bubonic plague. As historical sources illustrate, managing the outbreak of an unknown disease has already been challenged by balancing citizen's health and a functioning society. The second section questions the role played by Big Data in today's pandemic preparedness and discusses its promise for future public health intervention. To better illustrate this discussion, the last two sections are dedicated to two examples of contact tracing implementation. On the one hand, we explore China's Health Code App, which has become a compulsory management tool to relax lockdown measures.^11^ On the other hand, Switzerland recently opted out of a pan-European project to develop a contact tracing App because of disagreements on privacy issues.

## Premodern attempts to balance citizen's health and a functioning society

At the beginning of December 1347, 12 Genoese ships unloaded their goods in the port of Messina (Sicily, Italy). The merchant ships came from the Black Sea Coast. Soon, a terrible disease started spreading throughout Italy. People got bumps in the groin, in armpit areas, and began to spit blood under extreme pain. Their skin was covered with dark spots because of internal bleeding. A few days later, they died of the Black Death, as the bubonic plague was called back then. Marseille was the first French city to be reached by the Black Death. From there, it spread northwards and reached the English coast near Southampton in December 1348. One year later, almost all of Europe was affected.^12^ For the people of the late Middle Ages, this epidemic was a catastrophe. Until today, there is no conclusive evidence that the bubonic plague was solely transmitted by rat fleas. Recent modelling attributes a potential role to pneumonic and/or human ectoparasites transmission.^13,14^ Back then, savants referred to pestilential miasma caused by putrefaction processes as the trigger of the disease, which could be fought by isolation, fumigation, and disinfection.^15^ In search for the Black Death's origin, societal outsiders represented a most welcome scapegoat resulting, for example, in the persecution of Jews disparaged as “well poisoners” or “miasma distributors.”^16^

As Tognotti underlines in her lessons from the history of quarantine, some of the contemporary commercial centres began to explore a different strategy. One such centre was the Republic of Ragusa, today the city of Dubrovnik, Croatia. In 1377, the councillors of the Republic of Ragusa wanted to preserve economic activities which implied keeping the labour force in good health. By strictly isolating all newly arrived people and their goods on a nearby rocky island, today's Dubrovnik was among the first places to introduce quarantine. This initiative contradicted medical and religious ideas of the time, but it was the predecessor of practices that have become familiar, again, in 2020: self-isolation and social distancing.^17^

A century later, Georgius Agricola (1494–1555), humanist, mineralogist, metallurgist, and (rather involuntarily) city physician of the commercial town of Chemnitz in Saxony (today Germany), was commissioned by his city superiors to design a strategy to handle the outbreak of a plague that was ravaging the kingdom of Saxony (second wave of the bubonic plague, in 1552). He systematically isolated the infected individuals outside the gates of Chemnitz.^18^ Agricola described his successful method in one of his books entitled *De Peste libri tres*, outlining many elements of epidemiological considerations still valid today. Specifically, he recommended tracing contacts infected people had before falling ill, as well as isolating and observing them. Furthermore, he introduced the idea of a second phase with appropriate strategies in case the situation in the city should deteriorate. In addition to social distancing and restricting public life to its essentials, Agricola proposed the establishment of special medical centres, so-called lazarettos (a term later adopted into military jargon) providing both medical care and quarantine. His lazarettes had a limited capacity, and he considered them to be effective only in connection with curfews and a reduction in trade, thereby avoiding later and possibly much worse damage to the economy. His logistical concept even included a strategy to get the town out of a lockdown, slowly seeking to resume public life and economic activity. This exit phase of an epidemic, he wrote, should be based on surveillance and the self-monitoring of citizens, both in line with early modern state doctrine.^19^

These two historical examples show how the management of a contagious disease had to be carefully balanced between the protection of citizens and the continuity of economic activity. Similar considerations were made during the third wave of the bubonic plague that ravaged the world during the second half of the 19th century. While Europe was least affected, estimations rise to 15 million deaths for the densely inhabited India, Indonesia, and China,^20^ particularly the Chinese province of Yunnan.^21^ The bubonic plague struck Yunnan when the province already had to deal with violent uprisings by Muslim minorities. This civil unrest made it difficult for authorities to implement isolation measures and rules of social distancing. Therefore, they began to appeal to the population's experience with infectious diseases, with success, as Émile Rocher (1846–1924), a French member of the Chinese Imperial Customs Service, reported. Among these precautionary self-help-strategies were accounts of self-isolation, the purification of houses via fumigation, strict neighbourhood monitoring, and the observation of animal behaviours.^22^

This finally made it possible to stabilize the death rate at a—still high—level of 4% without putting even more pressure on the already weakened economy of the province.^23^ Balancing citizen's health and a functioning society means distributing possible damage in protecting as many lives as possible while preserving as much of the daily (economic) activities as possible. The art of epidemiological crisis management resembles therefore a delicate risk strategy. Although the tools to manage population behaviour have evolved since the Middle Ages, the careful weighing of the balance of control remains a tricky endeavour.

## Tracing contacts, changing our world

Before the outbreak of SARS-CoV-2, the modelling of pandemic preparedness scenarios largely depended upon only two sets of information: Data derived from surveying the spread of the common flu (influenza A-C) and incomplete information about historical pandemics such as the Spanish Flu (1918–1919), the Asian Flu (1957), the Hong Kong Flu (1968), or even other types of viruses such as HIV.^24^ Although most experts agreed that a pandemic was likely to occur in the future, some had doubts if one could ever be fully prepared for an outbreak of a hitherto unknown contagious disease. As Caduff^25^ points out, pandemic prophecy models the future with information of the past, an experiment on shaky ground. Even Google's attempt to develop pandemic influenza models based on current search terms has not been very successful.^26^ Nevertheless, digital reporting of confirmed infections, deaths, and recoveries created an avalanche of available information during the COVID-19 pandemic, which made news tickers never rest Relaxation of public health measures was tight to low infection rates requiring to identify infectious individuals early enough to break the chain of infection. Hence, digital contact tracing appeared as a ‘golden grail of mapping’ promising a fast and safe exit strategy out of the lockdown. According to Sy Pretorius, the Executive Vice President of Parexel, a global provider of biopharmaceutical services, humankind is entering an era of real-world evidence based on aggregated data sets.^27^ However, contact tracing data provided by smartphones are a decisive ingredient to achieve real-time public health management, and its implementation still faces security-relevant as well as structural challenges.^28^ Yet, the World Economic Forum sees digital monitoring as a tool to ideally balance economic damage and the protection of the population. Others, for example, Ericson Chan, the Chief Executive Officer of Ping An Technology (平安科技 ping’an keji) in China (until 2021), one of the world's largest service providers for finance and health institutions, are convinced that our societies will undergo a radical transformation. Chan predicted a new ‘social contract for data privacy’ for the post COVID-19 pandemic world.^29^ According to Chan's reasoning, such a new social contract already casts its shadow:

There is a growing number of countries like Israel, South Korea, China are combating COVID19 with rigorous contact tracing. How this is done is leveraging on everyone’s geolocation and online activity to subsequently monitor and control the spread of the virus. For now, how this will impact privacy is still uncertain. Yet, the fact is that it opens a whole new can of worms on data privacy under the humanitarian banner. Many governments are now insinuating that digital contact tracing will need to last for at least 2 years until a vaccine is fully available. AI and big data will also be more critical than ever before, especially for impact analysis and forecast Further, surveillance types of data will be used more widely, more frequently and in a more granular manner.^29^

Despite what Chan's quote might suggest, contact tracing and geolocation do not generate the same type of data, neither are their privacy risks comparable. Geolocation can reveal detailed movement profiles of individuals such as Marauder's map in the fictional world of Harry Potter. Geolocated data is for example produced when using Google Maps for orientation, to spot the next Uber, or optimizing jogging routes. What can be very useful for private purposes can become problematic under specific circumstances as shown by the case of the fitness App *Strava* which revealed the location of secret US army bases when soldiers shared their jogging routes online.

Contact tracing does not rely on the geographical location, but on smartphone-to-smartphone interaction via Bluetooth. In practice, contact tracing Apps are designed to scan for other nearby Bluetooth signals which are identifiable by a unique international mobile equipment identity (IMEI) number. If two smartphones come close enough, they save the IMEI number of the incoming Bluetooth signal and create a contact log with date and time of the interaction. When an individual tests positive for COVID-19 all users who had been close to the infected individual within a specific timeframe can then be alerted via the App and are usually recommended some days of self-isolation to break the chain of infection. Digital contact tracing seems less questionable regarding data protection and privacy compared to geolocation if its use remains voluntary. However, some concerns were expressed related to hacking after the Indian COVID-19 tracking App had been hacked within <4 h.^30^ What role the contact tracing App will play for the upcoming years of public health management or only for the final phase of the COVID-19 pandemic is subject to conjecture. Yet, what current developments show is that even the best efforts to prepare well for the next pandemic or prevent a second wave of COVID-19 must deal with two unknown variables: space and time.^31^ As it has already been the case for the first outbreak of SARS-CoV-19, local and temporal realities can be quite different. Although being a pandemic, thus a global issue, it has been addressed almost entirely on a national level. The most likely scenario will therefore depend on local solutions and negotiations in the respective sociocultural contexts, local probably standing for the entity of the national state. The following sections illustrate the diverging cases of China and Switzerland: while the Chinese government implemented a compulsory real-time monitoring based partly on geolocation to manage the spread of COVID-19, Swiss researchers are developing a decentralized contact tracing App that has been approved in late 2020 by the parliament.

## Tracing citizens as a real-time experiment: The health code (健康码 jiankangma) in China

The severe acute respiratory syndrome (SARS) epidemic in 2003 had taught the Chinese government the lesson of balance, the memory of the infectious disease leading to severe social and economic problems being still vivid. This partly explains the development of an efficient – albeit somewhat authoritarian – multicomponent strategy. The Chinese playbook on health hazards contains more than hundred measures: communication, education, hotline and telemedicine consultation, temperature examination, retroactive tracing, closing national and local borders, restriction in gathering and travelling, personal hygiene and protection gears, testing, self-quarantine and designated isolation facilities, temporary workforce and resource reallocation, financial support, free medical treatments, research, etc. Accompanying these rather traditional measures, China promoted digital empowerment (数字化赋能 shuzihua funeng) for governance, business, and population. Two novelties not only speeded up the containment process but supposedly fixed some shortcoming identified during the SARS crisis, namely the secretive approach of the government and the initial slack and uncooperative attitude of its local officials.^32^

First, China started in January 2020 to disclose real-time data about the outbreak (including infected cases, death toll, and recoveries) on main news outlet^33^ as well as on the front page of ubiquitous Apps such as Alipay for mobile payment and WeChat for instant messaging. Tech giant Baidu integrated a hyper-local live epidemic display of infected and recovered individuals near the user, with details down to the address and diagnostic timeframe into its navigation map App.^34^ Second, the government recognized formalism and bureaucratism as obstacles limiting coordination and effective mobilization.^35^ It is important to explain here that the institutional culture promotes flexibility in the local management of top-down guidelines, thus allowing trial and error until the best practice is found. This structural “directed improvisation” fosters local initiatives that, if proven efficient, would be applied nationwide.^36^

The Health Code App (健康码 jiankangma) was one of these local initiatives, developed as public–private cooperation between Zhejiang local government and Alibaba. With the objective to reassure people when resuming daily work in February, a close-contact detection App was developed to facilitate the risk assessment of having contracted the disease.^37^ Much has been published internationally about the two hospitals built in 10 days, but only a few have been reported about the contact tracing App being created by a team of 150 engineers in <48 h.^38^ The Health Code App – or more accurately Apps in the plural, as each province ended up developing its own version – assigned each individual a QR code which could adopt three traffic-light colours: green, the user is considered healthy, and access is unrestricted; yellow, a preventive 7 days isolation is required; red, 14 days quarantine is required. The Health Code served the same purpose as the systematic handwritten registration form granting access at a checkpoint, albeit in a convenient electronic version. Within one week, the Health Code App was adopted by 90% of the population in Zhejiang Province (children and elderly could be registered as companions of the main user), of which 98.2% were green. Such a broad acceptance is unsurprising as life without Health Code was impossible to the extent that even fugitives had to surrender.^39^

The fear of unpredictability and false-positive occasionally caused unease to a few individuals, as the system was not without glitches. The supporting hotline was overloaded by citizens asking for a correction. Yet, knowing that only green-rated citizens would dare to go out was reassuring for the Chinese population. Nevertheless, it is not public how exactly the colours are attributed to citizens.^40^ The Health Code App, often directly accessible on Alipay or WeChat, requires the user to sign up with name, ID number, and phone number. Rating is updated daily based on three types of data: (1) self-reported information on physical symptoms such a fever and cough, travel history, and contact with infected individuals (to manually input every 1 to 3 days); (2) provincial and national databases of confirmed infected cases (updated daily, flawed by the lack uniformly recognized standard); (3) record of punctual global positioning system (GPS)-based localization data sent when scanning the personal QR code (to retroactively assess close-contact with infected cases and to prevent from bypassing the system by using screenshots). Note that GPS and cellular data, down to 10–20 m accuracy, would be insufficient for effective contact tracing. Moreover, an analysis of the code by the New York Times indicates that Health Code might be more than just a real-time pandemic tracing system. According to their examination, the App is transferring data to the police, which could lead to an unprecedented case of social surveillance. Countless checkpoints with security guards were deployed at every entrance and strategic intersections through which control was ensured by scanning the QR Health Code, measuring temperature, disinfecting, and verifying the wear of protection masks. Residence compounds were secured by community-based surveillance, a volunteer-run informal system developed 1000 years ago,^41^ revived to monitor the respect of self-isolation measures. The example of the Health Code App shows how social surveillance does not only rely on technology, but to an important extent also on an impressive mobilization of human supervisors either paid by the state deployed by volunteering networks.

As a matter of fact, delegating pandemic containment to technologies only is impossible without a fair share of manual labour to develop, maintain, enforce, and complement the system. China seems to shift the weight more on the collective side of the balance of control, mostly ignoring privacy concerns. Chinese citizens seem to consider Health Code useful to deal with the pandemic, little being bothered by the protection of their data, only wishing that it will be of limited use. As observers, we can only hope that the parallel to Maurauder's map does not match too exactly, envisaging the Chinese authorities swearing “I solemnly swear that I am up to no good.” when implementing the Health Code. In every case, it remains to be seen if technology, by collecting a huge amount of data, could be a real game changer in the aftermath of the crisis when the time has come to fine-tune our scientific understanding of this epidemiologic and social phenomena.^42^

## Contact tracing in Switzerland: Wilhelm Tell with smartphone

In mid-April 2020, an international group of researchers published a “Joint Statement on Contact Tracing.”^43^ Their aim was to counteract a possible expansion of data use and pursue a decentralized approach towards data storage and processing, as provided by DP3T, PACT, or TCN encryption protocols. In contrast to this international focus, the European Parliament began to favourize another, more centralized option, by endorsing the Pan-European Privacy-Preserving Proximity Tracing consortium.^44^ This consortium turned quickly to more centralized solutions and finally removed the decentral DP3T security protocol, which was co-programmed by researchers from Switzerland from their homepage and GitHub resources. As a response to this, the EPFL epidemiologist Marcel Salathé and the ETH professor Kenneth Paterson left the European consortium.

In the following weeks, the teams around these two researchers intensified their efforts to further develop a local, decentralized, and Swiss solution based on a DP3T protocol. This solution is, as the freely accessible project documentation shows,^45^ not only centred around security, privacy, and openness goals, but also integrates educative components for the re-entry into the containment (or exit-phase) of the pandemic. Beyond its proximity tracing engine, the final App will also distribute official public health information and instructions for self-isolation in the case of exposure.

Such incentives for the user's self-control during the reopening phase of the country, mirror in their intended functionality Agricola's strategy from the 16th century to create an equilibrium between individual freedom, community health, political stability, and economic interests. While infection rates might rise again, during the different phases for the reopening of public life in Switzerland in May 2020, the App is designed to serve as a risk mitigation tool to reduce both the logistic burden on hospitals and the pressure on the Swiss economy due to a prolongated lockdown period. As such, the Swiss Federal Council hopes to use this or similar Apps during the reopening period to “avoid the declaration of emergency decrees” or even lockdown orders in future epi- or pandemics.^46^

While the “Swiss PT-App” is currently being tested in a field trial by 100 Swiss soldiers, its public release was initially planned for Friday 8 May 2020 but has later been postponed to June as the law requires approval by the parliament. The increased media and official reference to the App and its history of development shows^43,44^ that it will also include another function. This function is more symbolic and implicitly aims to contribute both to the strengthening of social cohesion and to the perceived agency of the population. Individual contribution to collective success is also frequently underlined in expert models, which suggest that 60% of the population should use a tracing App to handle the public health contingencies efficiently. It creates the illusion of a level of participation in crisis management that goes beyond state appeals for social distance or wearing protective masks in public. In view of the recent rise of protests against “stay at home orders,” “social distancing,” and “lockdown” in the economy (e.g. anticoronavirus lockdown protests in Bern, capital of Switzerland on 16 May 2020) technological tools may seem like a solution in the hands of the population. Given both their design and the way how they display epidemiological and political information, the user (even if passive) is at the centre of them. In the inherent narrative of contact tracing Apps, it is the user who exerts epidemiological control and can at the same time indulge the illusion of low-risk mobility in the context of the reopening of her/his/their economic and social life world. This regulatory illusion of participation might be the biggest difference between past methods of managing epi- and pandemics and those of today.

The “Swiss PT-App” might even go a step further than other proximity tracing solutions, since both the communication surrounding it and its presentation are charged with the symbolism of Swiss libertinism. This finds its reflection in a discursive focus on decentralization. It might be no accident that Marcel Salathé and his colleague Kenneth Paterson are elevated in various media narratives to modern Wilhelm Tell of the pandemic. An iconography that was by his contemporaries of the 16th century also applied to Agricola and his regulatory work “de peste.” Interestingly, the legal construct and the communication strategies around the “Swiss PT-App” fix a positive cultural value of libertinism on to this solution to safeguard the economy and public health. We notice that similar technologies proposed in China, even though they arise outside from top-down control of the government, are perceived as necessarily authoritarian by nature. Chances are good that the Swiss contact tracing solution will be approved by the Swiss parliament, but whether a sufficiently high proportion of Swiss citizens will adopt the App is more than debatable.

## Conclusion: Can citizens equilibrate the balance of control?

Managing a pandemic is not only about public health but also about the continuity of social and economic life. Strategies to balance the control of these two realms require careful considerations on an individual and a collective level. Commercial centres throughout history controlled human mobility to avoid long-term economic damage. Then and now, targeted contact tracing was preferred over a general lockdown of society. The smartphone becoming a common good in most industrial countries offered new technical solutions which promise to facilitate contact tracing. A feature that is specifically important in a world that is much more globalized than during the Middle Ages. Even if the epidemiological benefit of such contact tracing Apps is not yet fully clear,^47^ their development delivers another message too: the idea of a committed state that invites its population to act themselves to overcome the lockdown in an act of solidarity. As the management of the third wave of the bubonic plague in the Yunnan province, using a contact tracing App becomes the duty of a responsible citizen, either imposed as in China or as a voluntary commitment such as in Switzerland. Such a symbolic participation of users might be able to cushion some of the balance of control lost in the management of the COVID-19 pandemic.

Even irrespective the problems related to data protection, technical and structural challenges, digital contact tracing is not the flawless solution it is promoted as. Our article indicates that the balance between citizen's health and a functioning society is a delicate equilibrium that must be continually rebalanced. The complexity of individual and collective needs during a pandemic cannot simply be met through a digital solution, as the experiences of the last two years have shown.
